# Regulation of intracellular transition metal ion level with a pH-sensitive inorganic nanocluster to improve therapeutic angiogenesis by enriching conditioned medium retrieved from human adipose derived stem cells

**DOI:** 10.1186/s40580-020-00244-5

**Published:** 2020-10-16

**Authors:** Yeong Hwan Kim, Euiyoung Jung, Gwang-Bum Im, Yu-Jin Kim, Sung-Won Kim, Gun-Jae Jeong, Young Charles Jang, Kyung Min Park, Dong-Ik Kim, Taekyung Yu, Suk Ho Bhang

**Affiliations:** 1 School of Chemical Engineering, Sungkyunkwan University, Suwon, 440-746 Republic of Korea; 2 Department of Chemical Engineering, College of Engineering, Kyung Hee University, Yongin, 17104 Republic of Korea; 3 School of Biological Sciences, Georgia Institute of Technology, Atlanta, GA 30332 USA; 4 Parker H. Petit Institute for Bioengineering and Bioscience, Georgia Institute of Technology, Atlanta, GA 30332 USA; 5 Department of Biomedical Engineering, The Wallace H. Coulter, Georgia Institute of Technology, Atlanta, GA 30332 USA; 6 Department of Bioengineering and Nano-Bioengineering, Incheon National University, Incheon, 22012 Republic of Korea; 7 Division of Vascular Surgery, Samsung Medical Center, Sungkyunkwan University School of Medicine, Seoul, 06351 Korea

**Keywords:** Angiogenic paracrine factor, Inorganic nanocluster, Ischemia, Stem cell therapy

## Abstract

Cell therapy based on human adipose derived stem cells (hADSCs) is a known potential therapeutic approach to induce angiogenesis in ischemic diseases. However, the therapeutic efficacy of direct hADSC injection is limited by a low cell viability and poor cell engraftment after administration. To improve the outcomes of this kind of approach, various types of nanoparticles have been utilized to improve the therapeutic efficacy of hADSC transplantation. Despite their advantages, the adverse effects of nanoparticles, such as genetic damage and potential oncogenesis based on non-degradable property of nanoparticles prohibit the application of nanoparticles toward the clinical applications. Herein, we designed a transition metal based inorganic nanocluster able of pH-selective degradation (ps-TNC), with the aim of enhancing an hADSC based treatment of mouse hindlimb ischemia. Our ps-TNC was designed to undergo degradation at low pH conditions, thus releasing metal ions only after endocytosis, in the endosome. To eliminate the limitations of both conventional hADSC injection and non-degradable property of nanoparticles, we have collected conditioned medium (CM) from the ps-TNC treated hADSCs and administrated it to the ischemic lesions. We found that intracellular increment of transition metal ion upregulated the hypoxia-inducible factor 1α, which can induce vascular endothelial growth factor (VEGF) and basic fibroblast growth factor (bFGF) expressions. Based on the molecular mechanism, the secretion of VEGF and bFGF by ps-TNC treated hADSCs showed a significant improvement compared to that of untreated cells. Injecting the CM collected from ps-TNC treated hADSCs into the mouse hindlimb ischemia model (ps-TNC-CM group) showed significantly improved angiogenesis in the lesions, with improved limb salvage and decreased muscle degeneration compared to the group injected with CM collected from normal hADSCs (CM group). This study suggests a novel strategy, combining a known angiogenesis molecular mechanism with both an improvement on conventional stem cell therapy and the circumvention of some limitations still present in modern approaches based on nanoparticles.

## Introduction

Human adipose derived stem cell (hADSC) therapy is adopted for treating of various ischemic diseases [1–3], since hADSCs can secrete therapeutically effective angiogenic and anti-apoptotic growth factors [4]. Despite their potential practical applications of ADSCs in clinical trials are still limited due to their indefinite cellular behaviors, including undesired cell differentiation and poor cell engraftment [5, 6]. Conditioned medium (CM), the cell-free medium retrieved from cell cultures was suggested as a solution to overcome these limitations [7, 8]. Although previous studies have demonstrated the therapeutic potential and safety of CM [9–12], its low concentration of major growth factors is still a critical problem [13, 14]. Previous studies reported that the application of transition metal nanoparticles enhanced the therapeutic efficacy of stem cells [15–17]. Manganese (Mn) and copper (Cu) have been reported to regulate neuroendocrine differentiation and migration of ADSCs, respectively [17, 18]. However, these transition metals are cytotoxic and cause in vivo damages. Mn induces neuronal oxidative damage and causes Parkinson’s disease [19, 20], whereas high concentrations of Cu causes mitochondrial dysfunction and DNA cleavage [21, 22]. Therefore, it is necessary to develop a novel method to safely deliver metal ions in the cell, thus inducing the intended therapeutic effects only. A successful hADSC treatment to ischemic diseases majorly depends on the secretion of angiogenic paracrine factors in the lesion; therefore, if cytotoxicity could be circumvented, intracellular delivery of metal ions may lead to improved angiogenesis. Nanoclusters based on transition metals could be a novel solution to catalyze the secretion of the angiogenic paracrine factor in the CM.

**Fig. 1:**
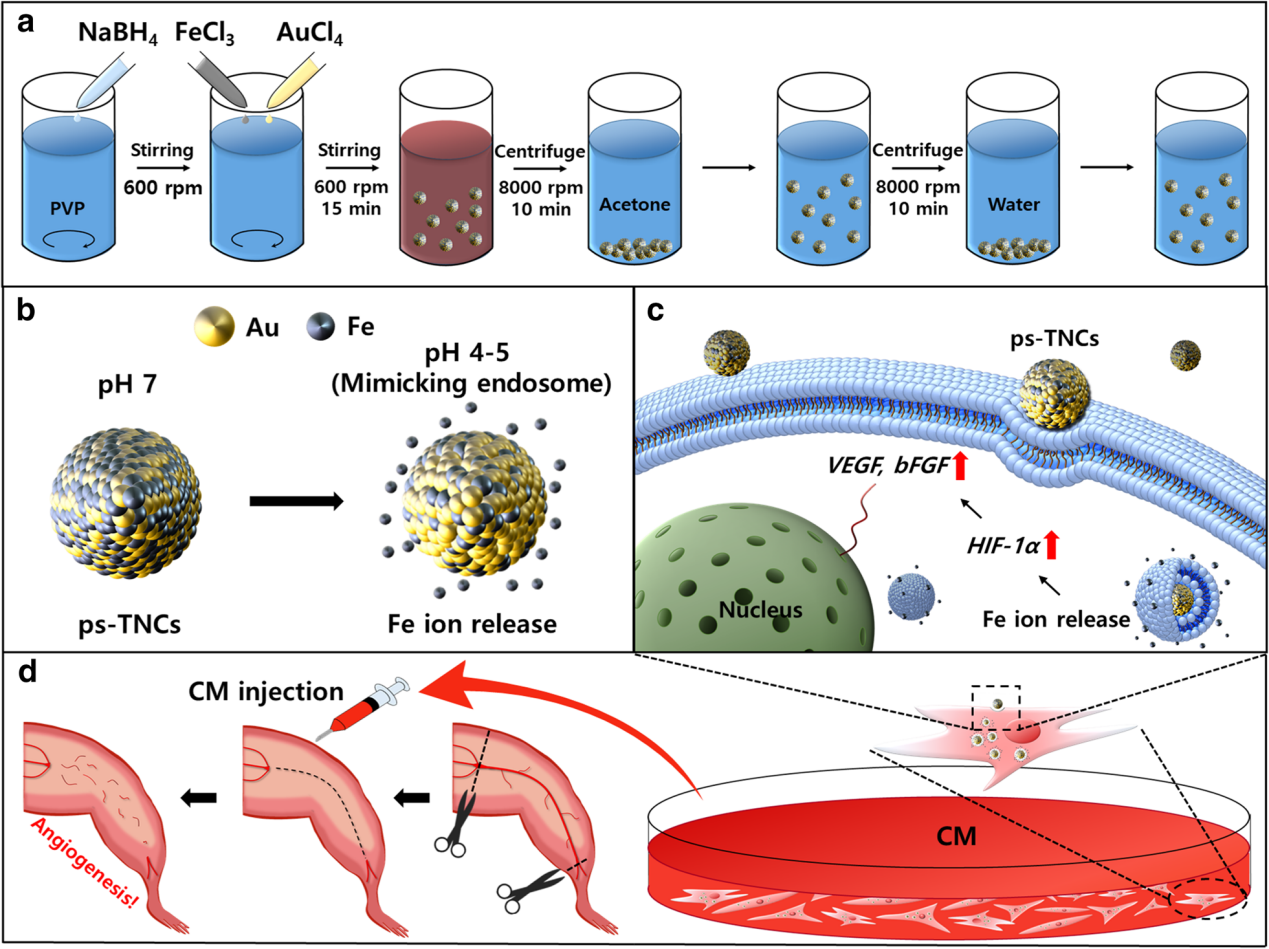
Schematic of ps-TNCs induced CM enhancement and therapeutic procedure. **a** Synthesis of ps-TNCs. **b** Release of Fe ions under low pH condition, which mimics hADSC endosomes. **c** Uptake of ps-TNCs through hADSCs endocytosis. **d** Therapeutic efficacy by in vivo enriched CM injection

In this study, we used novel pH-sensitive transition metal nanoclusters (ps-TNCs) that were designed to control the intracellular ion concentration (Fig. 1). Previously, iron (Fe) ions were known to induce hypoxia-inducible factor 1-alpha (*HIF-1α*), which can improve the therapeutic efficacy of stem cells [23]. Other papers have demonstrated that *HIF-1α* promotes the expression of angiogenic paracrine factor genes such as VEGF and bFGF [24, 25]. As the ps-TNCs used in our study can release transition metal (Fe) ions at pH condition resembling those of endosomes pH 4–5 [26], we hypothesized that our ps-TNCs could promote angiogenic paracrine factor secretions after endocytosis by hADSCs and subsequent molecular regulation (Fig. 1). We also hypothesized that the CM retrieved from ps-TNCs treated hADSCs, further enriched by angiogenic paracrine factors, could improve therapeutic angiogenesis compared to conventional CM injection. As gold (Au) is known to be non-toxic and non-biodegradable, it was selected to synthesize the ps-TNCs. For the same reason, Au nanoparticles were used to track intracellular localization of ps-TNCs; simple modifications to the surface of these nanoparticles may provide additional properties to further the angiogenic efficacy of hADSCs. After synthesizing ps-TNCs, the concentration of ps-TNCs was optimized to promote the angiogenic paracrine factor secretion while avoiding cellular damage to hADSCs by evaluating the in vitro cytotoxicity and *HIF-1α* gene expression. Thereafter, we evaluated the maintenance of promoted angiogenic paracrine factor secretion by hADSCs treated with ps-TNCs. As for in vivo comparation of angiogenesis, we injected CM retrieved from hADSCs treated with ps-TNCs (ps-TNCs-CM group) and conventional CM (CM group) in hindlimb ischemic mice. The angiogenesis improvement, often leading to hindlimb salvage in the ps-TNCs-CM group, was evaluated for 28 days on the basis of morphological imaging, microvessel gene expression, histological, and immunohistochemical analyses. Applying the optimized concentration of ps-TNCs to hADSCs significantly improved the angiogenic paracrine factor secretion to CM, though avoiding cytotoxicity or cell phenotype change. Enhanced *HIF-1α* expression by controlling intracellular Fe ion level with ps-TNCs was confirmed, and this improved angiogenic paracrine factor secretion. Hindlimb salvage, microvessel gene expression, histological, and immunohistochemical analyse showed marked improvements over the conventional CM group, while the ischemic tissue in the ps-TNC-CM group also had a dramatic increment of CD31^+^ and smooth muscle alpha (SM-α)^+^ microvessels. Our new CM enrichment strategy based on ps-TNCs showed successful angiogenesis compared to conventional method. We believe that this strategy might enhance a wide range of biomedical therapies centered around angiogenesis and CM injection.

## Experimental

### Synthesis of ps-TNCs

To synthesize ps-TNCs, 100 mg of polyvinylpyrrolidone (PVP, MW = 55,000 Da, Sigma-Aldrich, St. Louis, Missouri, USA) was dissolved in 9 mL of deionized (DI)-water and magnetically stirred (600 rpm, 15 min) at room temperature (25 °C). Aqueous solution (1 mL) containing 4 mg of NaBH_4_ (Sigma-Aldrich) was added to previously prepared PVP solution. Subsequently, 1 mL of an aqueous solution containing 4 mg of HAuCl_4_∙xH_2_O (99.995%, Sigma-Aldrich) and 1 mL of an aqueous solution containing 2 mg of FeCl_3_ (98%, Sigma-Aldrich) were added to the reacting solution, which was then stirred at 600 rpm for 15 min at room temperature. The final product was collected through centrifugation (8000 rpm, 10 min) and flushed two times with DI-water and propan-2-one, alternately. Synthesized ps-TNCs were dispersed in 200 mL of DI-water. To synthesize AuNPs, the same chemicals and conditions, but without FeCl_3_, were applied.

### Preparation of CM

Human ADSCs (1.6 × 10^6^ cells) were cultured in a 150-mm cell culture Petri dish (Corning Inc., Corning, New York, USA). After 24 h of incubation, the medium was changed to serum free DMEM with or without ps-TNCs, and then incubated for 12 h in a humidified incubator. After three PBS washes, the cells were moved to fresh serum-free DMEM, and cultured for 2 days. The CM was centrifuged at 1500 rpm for 5 min to eliminate cell debris.

### Terminal deoxynucleotidyl transferase dUTP nick and labeling assay

The terminal deoxynucleotidyl transferase dUTP nick and labeling (TUNEL) assay was performed to determine apoptotic activity in cultured hADSCs using an ApopTag® Fluorescein In Situ Apoptosis Detection Kit (Merckmillipore, Darmstadt, Germany) according to the manufacturer’s instructions. The hADSCs treated with serum free medium for 24 h served as a negative control group to demonstrate apoptosis. After TUNEL staining, fluorescence signals were detected with a fluorescence microscope (DMi8, Leica).

### Mouse hindlimb ischemia model

Four-week old female athymic mice (BALB/c-nu, 20–25 g body weight, Orient, Seongnam, Gyeonggi, Korea) were anesthetized with xylazine (10 mg/kg, Bayer, Seoul, Korea) and ketamine (100 mg/kg, Yuhan, Seoul, Korea). The femoral artery and its branches were ligated using a 6–0 silk suture (AILEE, Busan, Korea). The external iliac artery and all of the upstream arteries were then ligated. The femoral artery was excised from its proximal origin as a branch of the external iliac artery to the distal point from where it bifurcates into the saphenous and popliteal arteries. All animal treatments and experimental procedures were approved by the Institutional Animal Care and Use Committee of Sungkyunkwan University (No. SKKUIACUC-17-5-3-4).

### Treatment of hindlimb ischemia

Immediately after arterial dissections, the mice were randomly divided into four groups (n = 8 per group): normal, untreated, CM (DMEM [serum-]), and ps-TNC-CM (DMEM [serum-], ps-TNCs). The untreated group served as negative control. The groups treated with CM received a daily injection of 200 µL of the assigned medium into the gracilis muscle of the medial thigh for 4 days.

### Histological examination

Ischemic limb muscles retrieved 28 days post-treatment were embedded in optimal cutting temperature compound (O.C.T compound, SciGen Scientific Inc., Gardenas, California, USA), followed by freezing at − 22 °C and slicing into 10 µm-thick sections. Next, the sections were stained with hematoxylin and eosin (H&E) and Masson’s Trichrome staining (MT staining) to examine muscle degeneration, tissue inflammation and tissue fibrosis.

### Immunohistochemistry

For immunohistochemical staining, sections were obtained as described in the previous section, then stained with anti-CD31 antibodies (Abcam, Cambridge, UK) and anti-SM α-actin antibodies (Abcam). Fluorescein isothiocyanate-conjugated secondary antibodies (Jackson Immuno Research Laboratories, West Grove, Pennsylvania, USA) were used to visualize the signals. The sections were counterstained with DAPI and examined using a fluorescence microscope (DMi8, Leica).

### Statistical analysis

All quantitative data were expressed as the mean ± standard deviation. For the statistical analysis, the one-way analysis of variance was performed with the Bonferroni correction using Sigmaplot (version 12.5, Systat software, San Jose, CA, USA). A *p* value inferior to 0.05 was considered to be statistically significant.

## Results

### Characterization of ps-TNCs

**Fig. 2:**
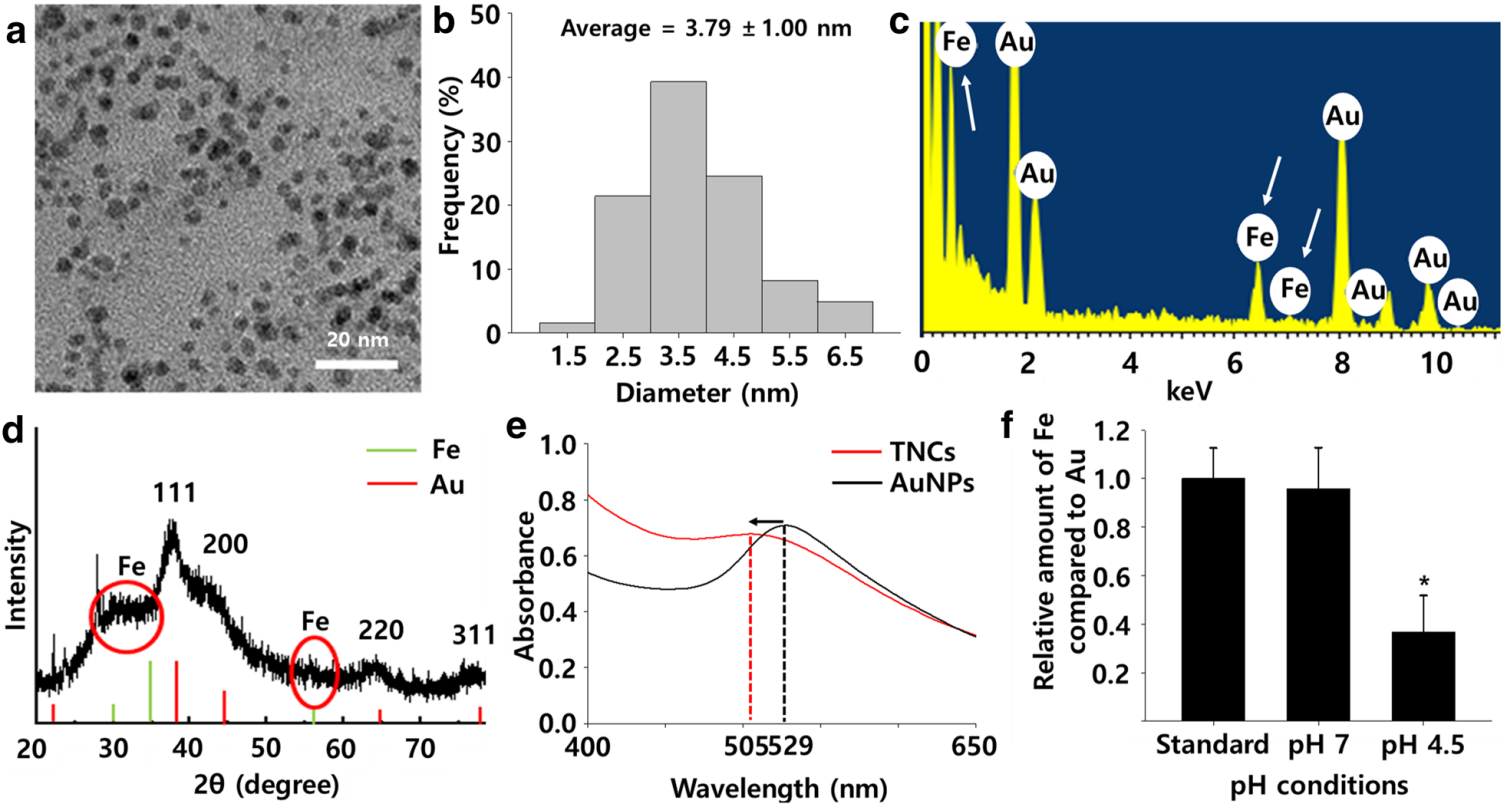
Characteristics of ps-TNCs. **a** TEM image of ps-TNCs. **b** Size distribution of ps-TNCs. The average size of ps-TNCs is 3.79 ± 1.00 nm. **c** EDX profile of ps-TNCs. White arrows indicate iron atom peaks. **d** XRD patterns of ps-TNCs. Red circles indicate iron ion patterns. **e** UV–vis spectra of gold nanoparticles (AuNPs, black line) and ps-TNCs (red line). Peak moves to left side due to the presence of iron. **f** Atomic ratio (gold/iron) of ps-TNCs according to the pH difference at 12 h as quantified by EDS. (n = 3, *p < 0.05 compared to other groups)

The ps-TNCs were synthesized in the presence of the stabilizer PVP by reducing Au^3+^ of HAuCl_4_ and Fe^3+^ of FeCl_3_ with sodium borohydride (NaBH_4_) for 15 min at room temperature. The color of injected HAuCl_4_ and FeCl_3_ immediately changed from mild yellow to dark red. TEM imaging of the ps-TNCs (Fig. 2a) showed spherical nanoclusters with an average size of 3.79 nm (Fig. 2b). EDX analysis demonstrated that transition metal (iron) was incorporated into the synthesized ps-TNCs (Fig. 2c). The XRD peaks are moved exiguously to higher angles compared to those of gold nanoparticles without any transition metal (iron) component (Fig. 2d), due to the size difference of the iron and the gold (Fig. 1c). A peak movement from 529 to 505 nm of plasmon resonance was observed by UV/Vis extinction spectra from an aqueous suspension containing ps-TNCs; it demonstrates a red shift between ps-TNCs and gold nanoparticles.[29]. Metal ion dissolution experiments of ps-TNCs were conducted under acidic and standard conditions (4.5 and 7) for 12 h (Fig. 2f). We have found that iron ions had dissolved out of the ps-TNCs in an acidic condition (iron/gold = 0.37), while the iron/gold ratio kept stable around 0.96 at pH 7.0. Metal ion dissolution tests showed that ps-TNCs could deliver iron ions to hADSCs intracellularly after endocytosis. Considering these results, we could conclude that the synthesized ps-TNCs were able to deliver transition metal (iron) ions to stem cells in a pH-sensitive manner.

### Optimizing the concentration of ps-TNCs for hADSC treatment

**Fig. 3:**
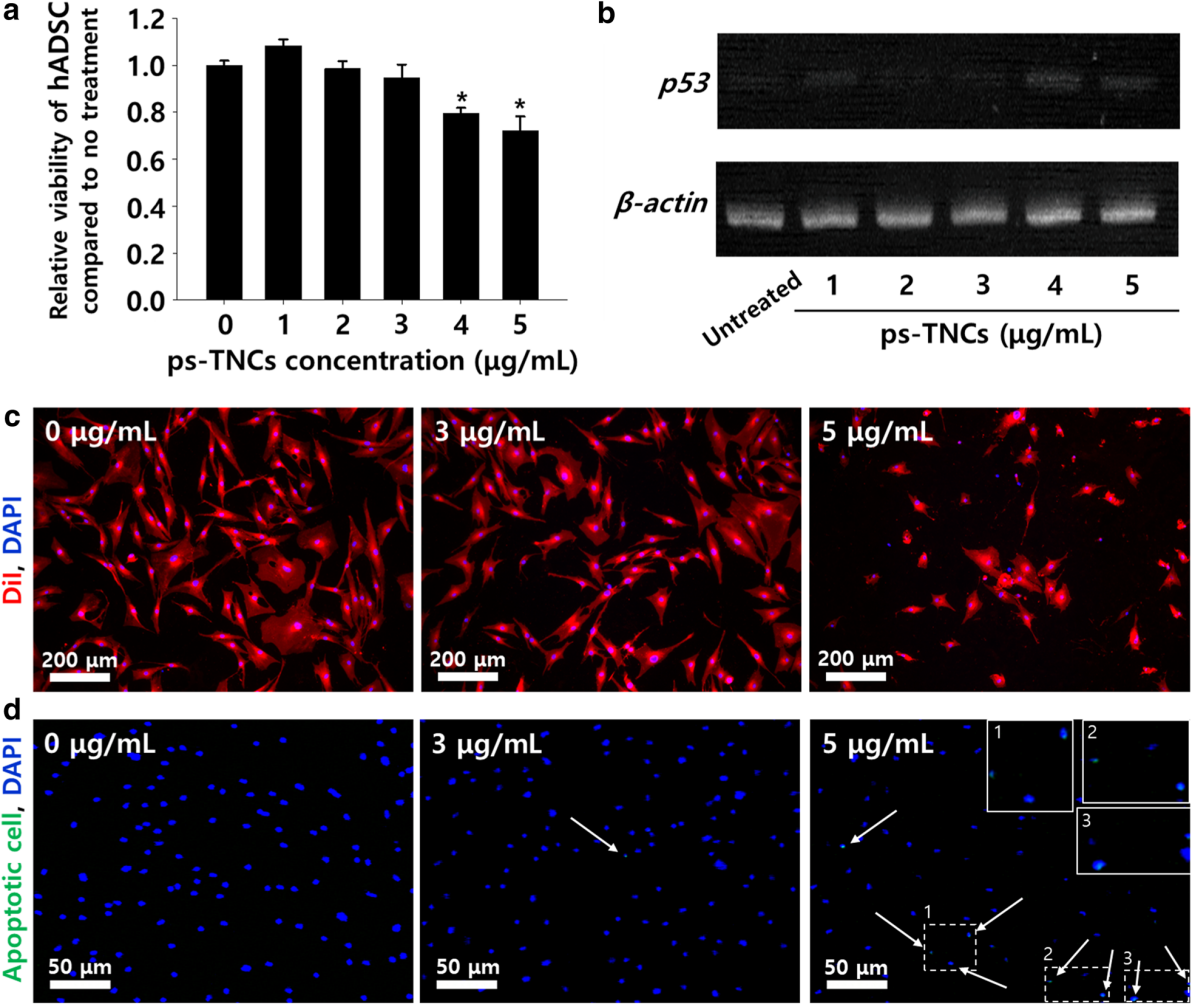
Optimizing the concentration of ps-TNCs for hADSC treatment. **a** Cell viability of hADSCs treated with various concentration of ps-TNCs (n = 4, *p < 0.05 compared to the untreated group). **b** Concentration-dependent pro-apoptotic mRNA (*p53*) expression in hADSCs. **c** DiI staining of hADSCs treated with ps-TNCs (red, cellular membrane). **d** TUNEL assay of ps-TNCs treated hADSCs (blue: nucleus, green: apoptotic cell)

The concentration of ps-TNCs for hADSC treatment was optimized (Fig. 3). In this study, in vitro cell viability was observed with CCK-8 analysis to identify the cytotoxicity of ps-TNCs to hADSCs (Fig. 3a). As shown in Fig. 3a, no statistical difference in cell viability was found between hADSCs (untreated) and with 3 µg/mL of ps-TNCs. The pro-apoptotic gene (*p53*) expression from hADSCs was significantly higher compared to that in the untreated group, as the concentration of ps-TNCs was increased over 3 µg/mL (Fig. 3b). Cellular morphology of hADSCs treated with ps-TNCs was observed using DiI staining (Fig. 3c). Significant morphological changes, cell shrink or circularization, were induced over 3 µg/mL of ps-TNCs treatment. TUNEL assay exhibited that treating with ps-TNCs at higher concentrations than 3 µg/mL, the number of hADSCs with high apoptotic activity was relevant and the total cell count decreased (Fig. 3d, white arrows). As a result, we have confirmed that the optimized concentration of ps-TNCs for hADSC treatment should not be more than 3 µg/mL.

### The effect of endocellularly released iron from ps-TNCs

**Fig. 4:**
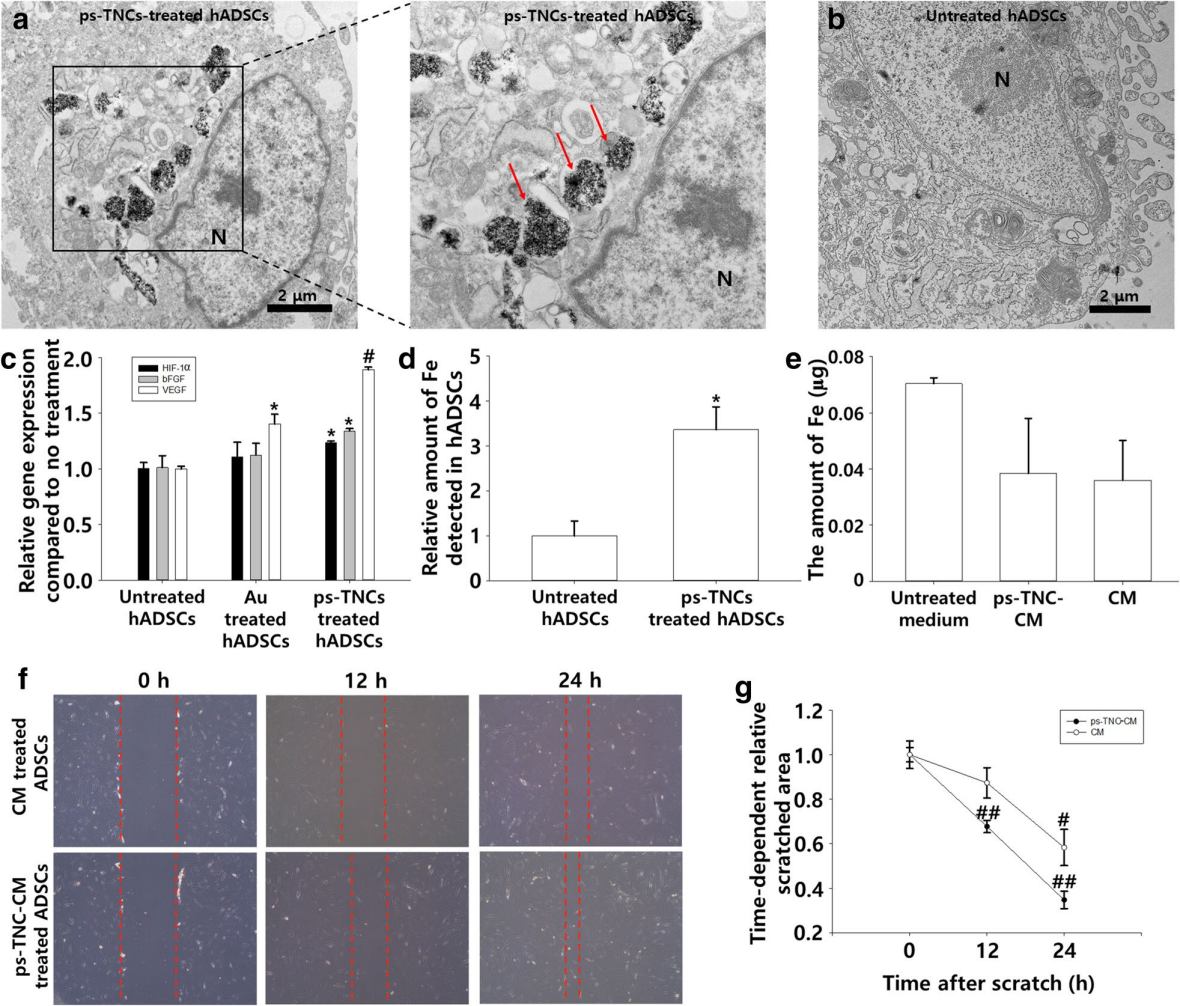
The effect of endocellularly released iron through endocytosis. TEM image of hADSCs (**a**) with and (**b**) without ps-TNC treatment (3 µg/mL, 12 h, N: nucleus, red arrows: ps-TNCs in endosomes, Scale bars = 2 µm). Dash lined box shows the high magnification image in the left side. **c** Comparison of *HIF-1a*, *hFGF* and *VEGF* expression in untreated hADSCs, with gold nanoparticles treatment (3 µg/mL, 12 h), and ps-TNC treatment (3 µg/mL 12 h, n = 3, #*p* < 0.01, *p < 0.05 compared to the untreated group). **d** Relative amount of iron ion detected in hADSCs after ps-TNC treatment as quantified by ICP-OES (n = 5, *p < 0.01 compared to the untreated group). **e** The amount of iron ion detected in various media as quantified by ICP-OES (n = 4). **f**, **g** The migration of hADSCs treated with CM or ps-TNC-CM for 24 h (n = 4, #*p* < 0.01, ##*p* < 0.001 compared to 0 h of each groups)

*HIF-1α* plays an important role in angiogenic gene expression [24, 25]. As demonstrated in Fig. 1, iron ions correctly delivered can upregulate the *VEGF* expression through *HIF-1α* regulation. To confirm whether our ps-TNCs can be delivered in hADSCs, we observed hADSC cytoplasm on TEM images to find the ps-TNCs delivered into the cells through endocytosis [30, 31] and then performed qRT-PCR (Fig. 4a). Relative expression of *HIF-1α*, *bFGF,* and *VEGF* was quantified with qRT-PCR; it was significantly higher in the group treated with ps-TNCs than in the group treated with gold nanoparticles (Fig. 4c). Relative amount of iron ions detected in hADSCs 12 h after ps-TNC treatment was evaluated by ICP-OES to be 4.05 µg/mL, which is approximately 3.4 times the amount in the untreated group. In contrast, significantly less of iron ions was detected in untreated cells (Fig. 4d). The amounts of iron ions contained in various media were quantified by ICP-OES. The amount of detected iron ions in untreated CM and ps-TNC-CM were not significantly different from untreated serum-free medium, which contains approximately 0.07 µg/mL of iron ions (Fig. 4e). The migration ability of hADSCs treated with CM or ps-TNC-CM was confirmed for 12 h and 24 h after scratch (Fig. 4f, g). hADSCs treated with ps-TNC-CM were recovered significantly wider area compared to CM treated hADSCs in both times. Irons were released in hADSCs through endocytosis; they endue migration ability to CM and few amount of them were detected in CM.

### Enhanced angiogenic potency induced by ps-TNCs in hADSCs

**Fig. 5:**
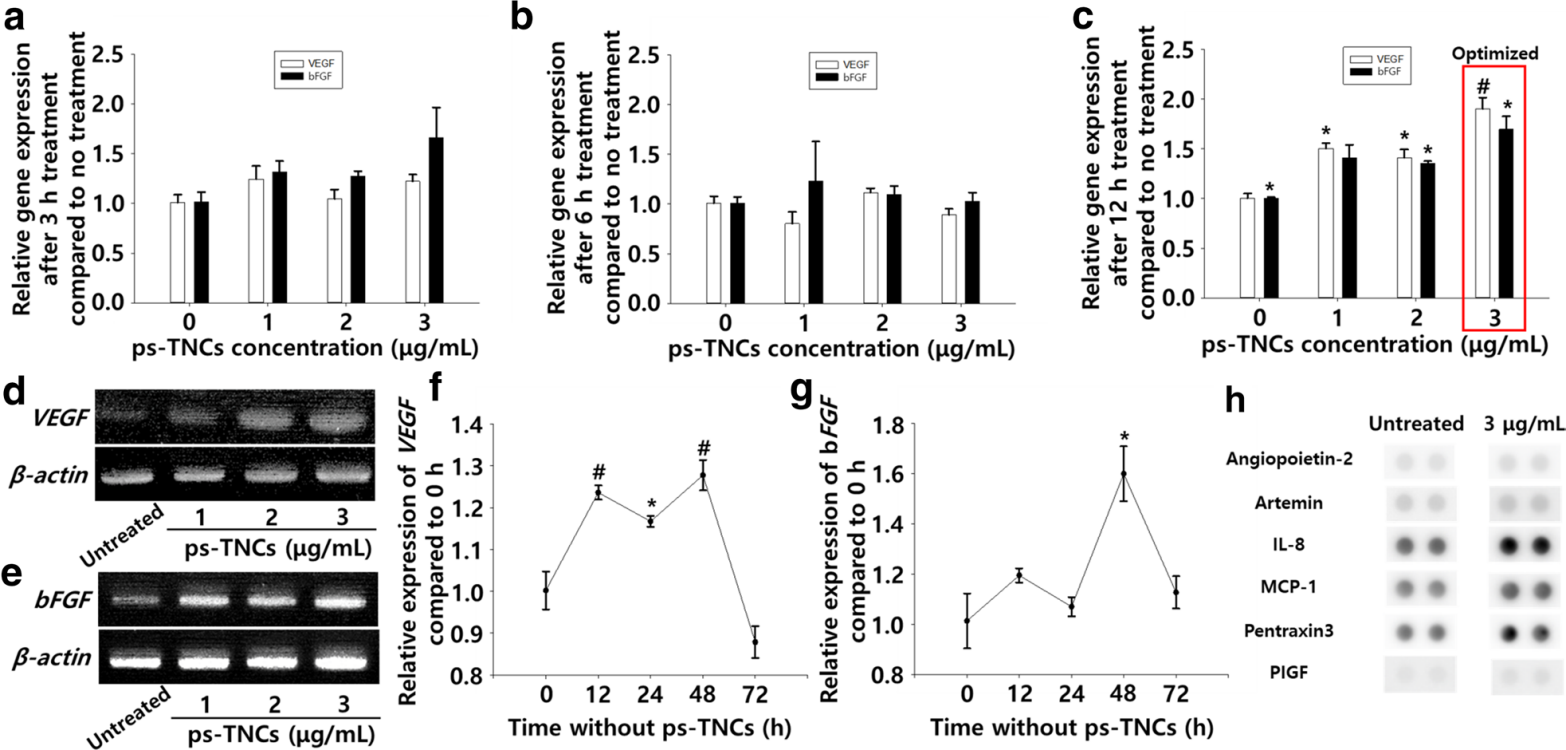
Enhanced angiogenic potency of hADSCs treated with ps-TNCs. **a**–**c** Concentration-dependent angiogenic gene expression with different treatment times, quantified by qRT-PCR. (n = 4, *p < 0.05, #*p* < 0.01 compared to the untreated group). Concentration-dependent (**d**) *VEGF* and (**e**) *bFGF* gene expression evaluated by RT-PCR. Duration of (**f**) *VEGF* and (**g**) *bFGF* expression in hADSCs after ps-TNC treatment for 12 h (n = 3, #*p* < 0.01 and *p < 0.05 compared to the untreated group). **h** The profile of angiogenesis-related protein in conditioned medium using human angiogenesis antibody array

We examined the gene expression of angiogenic paracrine factors using both RT-PCR and qRT-PCR. The concentration of ps-TNC and the time dependent angiogenic gene expression in hADSCs were quantified by qRT-PCR (Fig. 5a–c). As shown in Fig. 5c, compared with the untreated hADSCs (serum-free medium, 12 h), the significant increase of *VEGF* expression was confirmed in 3 µg/mL of ps-TNCs treated group (serum-free medium, 12 h). *VEGF* expression in hADSCs treated with 1 or 2 µg/mL of ps-TNCs (serum-free medium, 12 h) also showed a significant increment compared with the untreated group. However, when hADSCs were treated with 3 µg/mL of ps-TNCs, *VEGF* expression was highest. A ps-TNC treatment of hADSCs for less or more than 12 h showed no statistical difference among the groups. As shown in Fig. 5b, *bFGF* expression was also improved when ps-TNCs (3 µg/mL) were applied to hADSCs compared with the untreated group (serum-free medium, 12 h). Similar to gene expression profile of *VEGF*, *bFGF* expression profile after a 12 h treatment with ps-TNCs (1 or 2 µg/mL, serum-free medium) also increased. However, the highest *bFGF* expression profile was confirmed in hADSCs treated with 3 µg/mL of ps-TNCs. Treatment longer than 12 h also induced downregulation of *bFGF* expression. The treated hADSCs showed a 1.90-fold increment in *VEGF* expression and a 1.70-fold increment in *bFGF*. RT-PCR results also confirmed the qRT-PCR results under identical conditions (12 h, 3 µg/mL, serum-free medium, Fig. 5d, e). After treatment, we also observed the duration of the improved angiogenic paracrine factor secretion (Fig. 5f, g). Human angiogenesis related proteins in CM and ps-TNC-CM were confirmed using human angiogenesis antibody array (Fig. 5h). Several pro-angiogenic proteins such as angiopoietin-2, artemin, IL-8, MCP-a, pentraxin3, and PIGF were detected more in ps-TNC-CM compared to CM. Without any additional gene transfection or drug treatment, the treated hADSCs maintained their improved *VEGF* gene expression for 72 h and *bFGF* gene expression for more than 72 h after removing ps-TNCs.

### Improved angiogenesis and limb salvage by ps-TNC-enriched hADSC CM

**Fig. 6:**
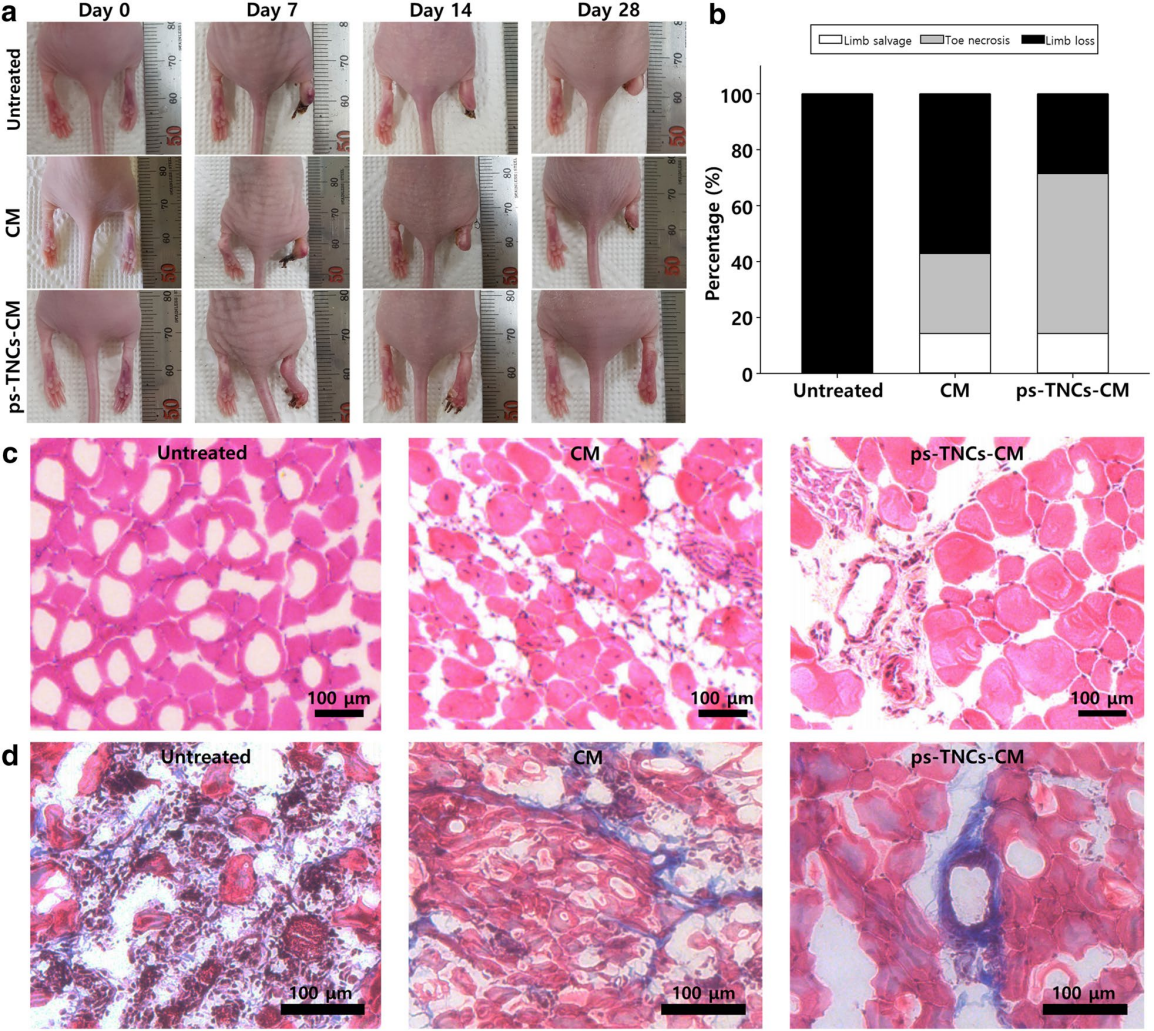
Increased limb salvage ratio with decreased inflammation and muscle degeneration in ps-TNC-CM group 28 days after the treatment (n = 7). **a** Representative images of ischemic limb after the various treatments. **b** Improved limb salvage ratio in ps-TNC-CM group compared to other groups 28 days after the treatment. **c**, **d** Representative histological images (H&E staining and MT staining) of ischemic lesions after the different treatments

**Fig. 7:**
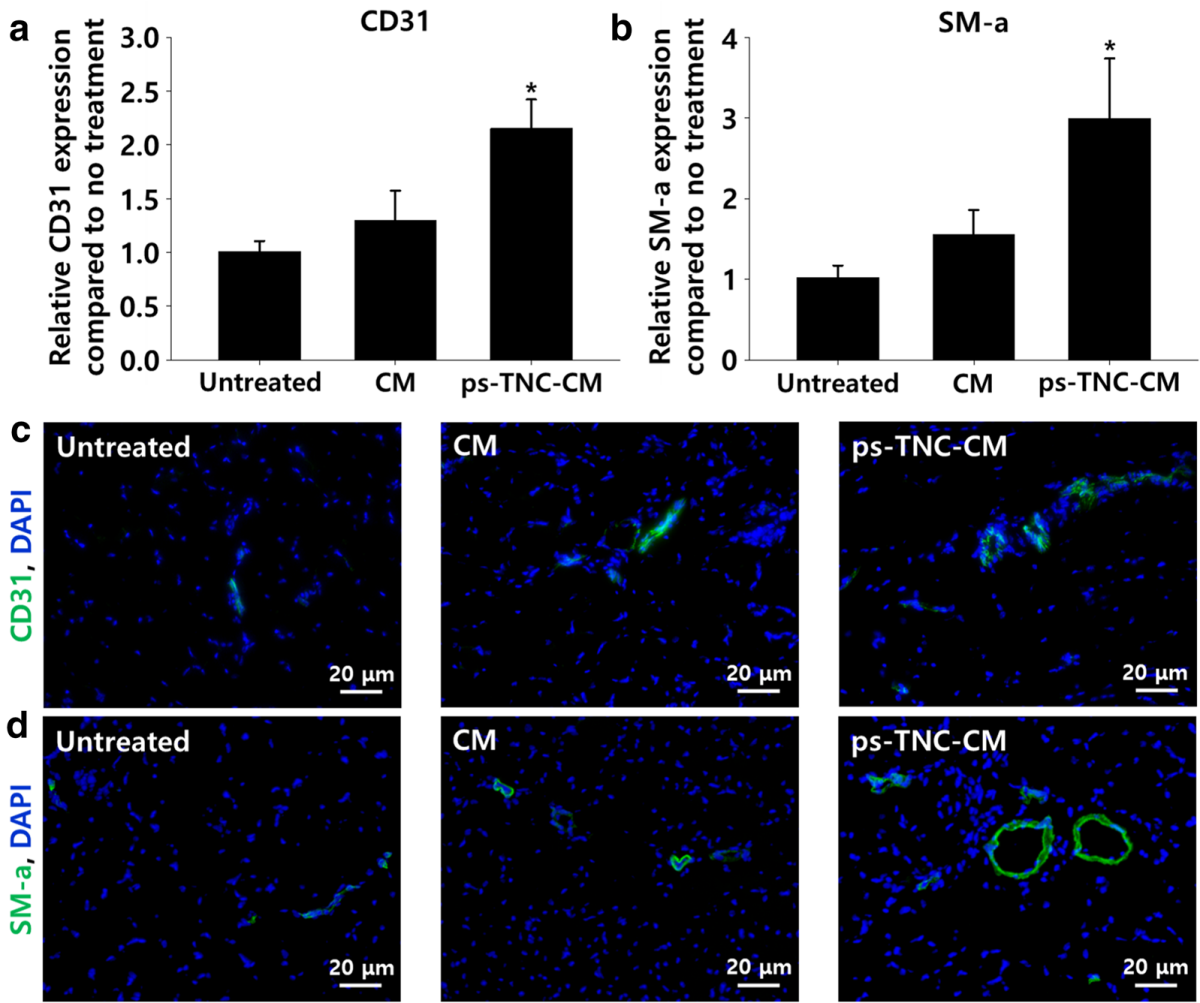
Improved angiogenesis induced by ps-TNC-CM in a mouse hindlimb model 28 days after the treatment. **a**, **b** Analysis of representative vascular genes (CD31 and SM α-actin) expression by qRT-PCR near ischemic lesions (n = 3, *p < 0.05 compared to other groups). **c** Immunostaining of CD31 (green) and **d** SM α-actin (green) in ischemic lesions. Blue indicates the nuclei (DAPI)

The morphological difference in ischemic lesions at various time points was evaluated according to the level of tissue necrosis and limb loss. Twenty-eight days after CM or ps-TNC-CM injection, tissue necrosis and limb loss in the ps-TNC-CM group were significantly different from those in other groups (Fig. 6). The number of mice with tissue necrosis, limb loss, and limb salvage in the CM group showed a significant difference compared to the untreated group; however, limb loss, which is the worst prognosis, was still frequently observed (Fig. 6b). H&E staining and MT staining of tissue collected from the ischemic lesion at day 28 showed the reduced muscle degeneration, inflammation, and fibrosis in the ps-TNC-CM group (Fig. 6c, d). The untreated and CM groups showed higher muscle degeneration, inflammation, and fibrosis in the ischemic lesions, compared with the ps-TNC-CM group. To evaluate the improved angiogenesis in the ischemic tissues, micro blood vessels in the lesions were quantitatively evaluated by qRT-PCR (Fig. 7a, b) and also immunostained with anti-CD31 and anti-SM- α antibodies (Fig. 7c, d). As indicated in Fig. 7, the highest gene expression of CD31 and SM-α was detected in the ps-TNC-CM group. Additionally, the highest number of CD31^+^ and SM-α^+^ micro blood vessels was detected in the ps-TNC-CM.

## Discussion

Adult stem cell therapies are considered as promising candidates for ischemic tissue regeneration through angiogenesis. However, cell viability and engraftment are usually low because of the severe microenvironment of the ischemic lesions. Thus, direct stem cell administration often has scarce therapeutic effects, poorly inducing angiogenesis and ischemic tissue repair. Nanoparticles are attractive tools for therapy [32–34]. Several studies have demonstrated that nanoparticles can improve the angiogenic efficacy of stem cells by enhancing the cell viability and paracrine factor secretion to overcome the conventional problems described. Despite their advantages, the high average half-life of nanomaterials remained as a potential risk to solve. In this study, we developed a pH-sensitive inorganic nanocluster that can control intracellular transition metal ion levels through a pH-responsive degeneration. Transition metal (iron) ions can be released from the nanocluster due to the low pH of hADSC endosomes, directly after endocytosis. Figures 2 and 3 showed that ps-TNCs can be applied to hADSCs to control the level of intracellular transition metal (iron) ions without causing cytotoxicity. Through in vitro experiments, we were able to select a suitable concentration range of ps-TNC. Iron has a relatively low standard redox potential compared to gold [35, 36]. Under low pH conditions found in hADSC endosomes, iron exhibits higher reactivity with hydrogen ions than gold, since the redox potential and chemical reactivity of iron and gold are clearly different. Therefore, iron in ps-TNCs can react faster and forms iron ions after endocytosis, while gold in ps-TNCs maintains its stable chemical structure.

The final goal of this study was to synthesize ps-TNCs as both regulators of transition metal ion levels and inducers of therapeutic angiogenesis in ischemic lesions. Previous nanomaterials, especially transition metal-based nanoparticles, have been synthesized and applied as tools for bioimaging or drug and gene delivery carriers [37, 38]. Unlike previous approaches, in our study, ps-TNCs were synthesized and applied to hADSCs as regulators of the intracellular transition ion level. As shown in Figs. 4 and 5, the ions released from ps-TNCs upregulated the *HIF-1α* gene in hADSCs and subsequently incremented *VEGF* and *bFGF* expression. The increment of *HIF-1α* gene expression in hADSCs might be related to the intracellular reactive oxygen species (ROS) level [39]. Previously iron ions have been reported to have interaction with mitochondrial membrane and upregulate ROS generation [40]. Since the ROS is known to upregulate *HIF-1α* gene expression, representative *HIF-1α*-downstream angiogenic factor such as VEGF might have been also upregulated. We proved that the injection of CM derived from hADSCs treated with ps-TNCs can induce angiogenesis and tissue regeneration in ischemic lesions. Additionally, injecting this enriched CM can be a solution to circumvent the actual hurdles encountered with stem cell administration. Previous studies have shown that hADSC administration can improve angiogenesis; the underlying molecular mechanisms majorly depend on the epithelialization of hADSCs and their secretion of angiogenic paracrine factors. All drawbacks of this approach considered, our novel method avoids many problems and is rather focused, as it targets micro vessel structure. Since intracellular release of iron ions induced a higher production of VEGF and bFGF in hADSCs, the administration of this CM enriched with growth factors at higher concentration could improve angiogenesis, also avoiding the problems encountered with the classical CM treatment, such as low cell viability and poor engraftment.

Our ps-TNCs promoted the expression of angiogenic paracrine factors in hADSCs (Figs. 4, 5), which enhanced the expression of the *HIF-1α, VEGF*, and *bFGF* genes. Treatment with 3 µg/mL of ps-TNCs highly upregulated *VEGF* an *bFGF* expression more than 72 h (Fig. 5). Angiogenesis related human proteins were confirmed in ps-TNC-CM (Fig. 5). Angiogenesis in ischemic lesions usually requires high supplementary concentrations of VEGF and bFGF, since both have been reported to show anti-apoptotic and angiogenic effects. Injecting ps-TNC-CM to the ischemic lesions might inhibit the apoptosis of the tissues, also supplying highly concentrated angiogenic paracrine factors with successive administrations. Indeed, ps-TNC-CM treatment showed improved in vivo angiogenesis and limb salvage. Shown in Figs. 6, 7, the ps-TNC-CM significantly raised limb salvage and the number of newly generated CD31^+^ and SM-α^+^ microvessels in the ischemic lesions. This enhanced in vivo angiogenesis may have been caused by the enriched angiogenic paracrine factors (VEGF and bFGF); the angiogenesis may have then brought blood flow to the ischemic lesions, increasing the limb salvage ratio and restoration of tissue.

In this study, we showed that ps-TNCs can improve the angiogenic efficacy of hADSCs by controlling the intracellular transition ion level. Furthermore, we have applied to ischemic lesions the CM derived from ps-TNC treated hADSCs, thus showing an improvement on therapeutic angiogenesis and overcoming some inveterate limitations of cell therapy and non-degradable nanomaterials. We believe that our ps-TNC based strategy can be applied to a wide range of biomedical problems related to angiogenesis and ischemic tissue regeneration.

## Conclusions

We have developed a pH-sensitive nanocluster based on gold and on a transition metal for the intracellular delivery of iron ion. Iron ions can now be successfully delivered by ps-TNCs into hADSCs through endocytosis at a low pH, resembling endosomal conditions. The expression of the angiogenic paracrine factors was enhanced by our method based on ps-TNC treated hADSCs, which upregulates the HIF-1α molecular pathway. As a result, injecting CM retrieved from hADSCs treated with ps-TNCs significantly improved therapeutic angiogenesis in the hindlimb ischemic mouse model compared to conventional CM injections. Our ps-TNC based CM therapy may provide a safe and innovative strategy for treating ischemic diseases.

## Supplementary information

**Additional file 1:** Additional experiment.

## Data Availability

All data generated or analyzed during this study are included in this published article and its Additional file 1.

## Abbreviations

Au: Gold
Fe: Iron
ps-TNC: PH sensitive-transition metal nanocluster
CM: Conditioned medium
VEGF: Vascular endothelial growth factor
bFGF: Basic fibroblast growth factor
IL-8: Interleukin-8
MCP-1: Monocyte chemoattractant protein-1
PIGF: Placental growth factor
CD31: Cluster of differentiation 31
SM-α: Smooth muscle- α
