# Supplementary material for: Regulation of intracellular transition metal ion level with a pH-sensitive inorganic nanocluster to improve therapeutic angiogenesis by enriching conditioned medium retrieved from human adipose derived stem cells
Source: Nano Converg. 2020 Oct 16;7:34. doi: 10.1186/s40580-020-00244-5 (PMC7567771; doi:10.1186/s40580-020-00244-5)
Supplement: Supplementary file 1 — Additional file 1: Additional experiment. [file 40580_2020_244_MOESM1_ESM.docx]

**Additional Experimental**

1.1. Characterization

Transmission electron microscope (TEM) images and energy dispersive X-ray (EDX) spectrum were captured using a field emission electron microscope (JEM-2100F, JEOL, Tokyo, Japan) operating at 200 kV. Powder X-ray diffraction (XRD) patterns were obtained with X-ray diffractometer (D-MAX/A, Rigaku, Tokyo, Japan) at 35 kV and 35 mA. The ultraviolet/visible (UV/Vis) spectra in the range of 250-850nm were recorded using a UV/Vis spectrophotometer (Cary 60 UV–vis, Agilent Technologies, Santa Clara, CA, USA). A direct reading of the elemental ratio of ps-TNCs was done with an inductively coupled plasma (ICP) spectrometer (Direct Reading Echelle ICP, Teledyne Leeman Labs, Hudson, USA).

1.2. Fe ion release from ps-TNCs under acidic condition

The amount of Fe ions released from ps-TNCs under different pH condotions was determined with the EDX spectrum recorded by the UV/vis spectrophotometer. pH 4.5 can be compared to the microenvironment of an endosome [26], while pH 7.0 was considered as the neutral condition of cell culture medium. Hydrochloric acid (HCl, 37%, Sigma-Aldrich) was added to a phosphate buffered saline solution (PBS, Gibco BRL, Gaithersburg, USA) to adjust the two different pH conditions (pH 4.5 and 7.0). The ps-TNCs were then dispersed in PBS at different pH conditions (pH 4.5 and 7.0) at room temperature. Each sample was centrifuged at 8000 rpm for 10 min to separate the nanoclusters from PBS. The changed Au/Fe ratio of nanoclusters was analyzed with EDX to confirm Fe release. Releasing time was confirmed according to gene expression results.

1.3. Human ADSC culture

hADSCs were purchased from Lonza (Bazel, Switzerland) and cultured in Dulbecco’s Modified Eagle Medium (Gibco BRL) supplemented with 10% (v/v) fetal bovine serum (FBS, Gibco BRL), and 1% (v/v) penicillin/streptomycin (Gibco BRL). The cells were cultured at 37°C in a humidified incubator with 5% (v/v) CO_2_ in air. The medium was changed every 2 days. Due to the possible interaction between nanoparticles surface charges and protein contents in the serum [27], a serum-free medium was used for the treatment on hADSCs.

1.4. Cellular uptake of ps-TNCs

The intracellular presence of ps-TNCs in hADSCs was quantified by measuring the amount of gold and iron in the cells. After 24 h of cell seeding onto 6-well plates, the medium was changed to ps-TNCs enriched serum free medium. After incubation, the cells were washed and lysed in nitric acid hydrochloride (mixture of nitric acid and hydrochloric acid in a molar ratio of 1:3) to dissolve all the components including ps-TNCs. Ionized samples were diluted with DI-water (1:4 (v/v)). The gold and iron concentrations were determined using an inductively coupled plasma optical emission spectrometer (ICP-OES, Varian, Palo Alto, CA, USA). To observe their ps-TNCs uptake, hADSCs were seeded on a 6-well plate and incubated with nanoclusters. Ultrathin sections of the cells were analyzed using TEM (Talos L120C, Thermo Fisher Scientific, Waltham, Massachusetts, USA) at 120 kV to observe the distribution of ps-TNCs. Briefly, the treated cells were washed three times with PBS to eliminate excess unbound ps-TNCs. Cells were treated with trypsin and washed three times with PBS, and fixed with Karnovsky’s fixative (5% glutaraldehyde (Sigma-Aldrich) + 4% formaldehyde in 0.1 M cacodylate buffer (Sigma-Aldrich) + 50 mg CaCl_2_ (Sigma-Aldrich)/100 mL H_2_O) for 2 h. Fixed cells were washed three times with 0.05 M sodium cacodylate buffer (Sigma-Aldrich). Post-fixation staining was performed using 2% osmium tetroxide (Sigma-Aldrich) in 0.1 M cacodylate buffer for 2 h at 4 °C. The samples were dehydrated in alcohol (30%, 50%, 70%, 80%, 90%, and 100% ethanol) and treated twice with propylene oxide (Sigma-Aldrich) for 10 min, then treated with propylene oxide and Spurr’s low viscosity resin for 2 h. Samples were further treated for 24 h with pure resin and embedded with it in molds. Resin blocks were polymerized at 70 °C for 2 days, then cut in ultrathin sections (70 nm) using Reichert Jung Ultracut (Reichert-Jung, Wien, Austria). The sections were stained with 1% lead citrate and 0.5% uranyl acetate and analyzed [28]. Cellular uptake time was confirmed on the basis of gene expression result.

1.5. Cell viability assay

Cell viability assay was determined with Cell Counting Kit-8 (CCK-8, Dojindo Molecular Technologies, Inc., Rockville, MD, USA), which measures the amount of formazan dye that is reduced by the intracellular dehydrogenase activities. The number of living cells is proportional to the amount of the formazan dye. Briefly, the hADSCs (1ⅹ10^4^ cells/well) were cultured for 24 h on 24-well plates with various concentrations of ps-TNCs, followed by rinsing with PBS three times. After replenishing the wells with fresh medium, the CCK-8 solution was added into each well and incubated for 2 h. Next, the absorbance at 450 nm was measured using a plate reader (Infinite F50, Tecan, Zurich, Switzerland). The cell viability was calculated as the percentage of viable cells relative to the ps-TNCs untreated cells (*n* = 4 per group).

1.6. Cell morphology assay

Cell morphology was evaluated by 1,1'-dioctadecyl-3,3,3',3'-tetramethylindocarbocyanine perchlorate (DiI, Thermo Fisher Scientific) staining. After cell treatment with the various concentrations of ps-TNCs for 24 h, they were moved to culture medium enriched with DiI (6.25 µM); the cells were incubated for 2 h at 37 °C and washed twice with PBS. The cells were fixed with PFA 4% solution for 10 min and washed again with PBS. After 4,6-diamidino-2-phenylindole (DAPI, Thermo Fisher Scientific) staining, DiI fluorescence was measured with a fluorescence microscope (DMi8, Leica, Wetzlar, Germany).

1.7. Reverse transcription-PCR (RT-PCR)

Samples were lysed in TRIzol reagent (Invitrogen, Waltham, Massachusetts, USA). Total RNA was extracted with chloroform (Sigma-Aldrich) and precipitated with isopropanol (Sigma-Aldrich). After supernatant removal, the RNA pellet was washed with 75% (v/v) ethanol, air-dried, and dissolved in 0.1% (v/v) diethyl pyrocarbonate-treated water (DEPC-treated water, Sigma-Aldrich). RNA concentration was determined by measuring the absorbance at 260 nm using a spectrophotometer. Reverse transcription was performed using 10 µL of 2ⅹEasy Tag SuperMix (TransGen Biotechnology, Beijing, China), 0.5 µL of cDNA, 0.5 µL of each primer, and 8.5 µL of sterile pure H_2_O, followed by PCR amplification of the synthesized complementary deoxyribonucleic acid. PCR consisted of 35 cycles of denaturing (94 °C, 30 s), annealing (58 °C, 45 s), and extension (72 °C, 45 s), with a final extension at 72 °C for 10 min. PCR was followed by electrophoresis on a 2% (w/v) agarose gel (Sigma-Aldrich) and visualization by RedSafe^TM^ (iNtRON, Seongnam, Gyeonggi, Korea) staining. PCR products were analyzed using a gel documentation system (WGD039, Daihan Scientific, Seoul, Korea). β-actin served as an internal control. The primers used for RT-PCR are shown in following table.

Table 1. Sequences of RT-PCR primers

| Primer | Sequence | |
| --- | --- | --- |
| Human  β-actin | Forward | 5′-GCA CTC TTC CAG CCT TCC TTC C-3′ |
|  | Reverse | 5′-TCA CCT TCA CCG TTC CAG TTT TT-3′ |
| Human  VEGF | Forward | 5′-GCA GAA GGA GGA GGG CAG AAT-3′ |
|  | Reverse | 5′-ACA CTC CAG GCC CTC GTC ATT-3′ |
| Human  FGF-2 | Forward | 5′-CCA CCT ATA ATT GGT CAA AGT GG-3′ |
|  | Reverse | 5′-GGG AGA CAA GAA AAC ACA AAC T-3′ |
| Human  p53 | Forward | 5′-GAA CAA GTT GGC CTG CAC TG-3′ |
|  | Reverse | 5′-CCA GCT GCC CAA CTG TAG AA-3′ |

1.8. Quantitative real-time polymerase chain reaction (qRT-PCR)

qRT-PCR was used to quantify the relative expression levels of the gene encoding glyceraldehyde 3-phosphate dehydrogenase (GAPDH), VEGF, FGF2, HIF-1α, β-actin, cluster of differentiation31 (CD31), and smooth muscle-α (SM-α). The samples were lysed in TRIzol reagent (Invitrogen); total RNA was extracted with chloroform and precipitated with isopropanol. After the supernatant was removed, the RNA pellet was washed with 75% (v/v) ethanol, air-dried, and dissolved in 0.1% (v/v) DEPC-treated water. For qRT-PCR, the SsoAdvanced^TM^ Universal SYBR Green Supermix kit (Bio-Rad, Hercules, California, USA) and the CFX Connect^TM^ real-time PCR detection system (Bio-Rad) were used. The primers used for qRT-PCR are shown in following table.

Table 2. Sequences of qRT-PCR primers

| Primer | Sequence | |
| --- | --- | --- |
| Human  GAPDH | Forward | 5′-GTC GGA GTC AAC GGA TTT GG-3′ |
|  | Reverse | 5′-GGG TGG AAT CAA TTG GAA CAT-3′ |
| Human  VEGF | Forward | 5′-GAG GGC AGA ATC ATC ACG AAG T-3′ |
|  | Reverse | 5′-CAC CAG GGT CTC GAT TGG AT-3′ |
| Human  FGF2 | Forward | 5′-GAC GGC AGA GTT GAC GG-3′ |
|  | Reverse | 5′-CTC TCT CTT CTG CTT GAA GTT-3′ |
| Human  HIF-1α | Forward | 5′-CAG TTA CGT TCC TTC GAT CAG TTG-3′ |
|  | Reverse | 5′-TTT GAG GAC TTG CGC TTT CA-3′ |
| Mouse  β-actin | Forward | 5′-GGC TGT ATT CCC CTC CAT CG-3′ |
|  | Reverse | 5′-CCA GTT GGT AAC AAT GCC ATG T-3′ |
| Mouse  CD31 | Forward | 5′-CAA ACA GAA ACC CGT GGA GAT G-3′ |
|  | Reverse | 5′-ACC GTA ATG GCT GTT GGC TTC-3′ |
| Mouse  SM-α | Forward | 5′-CAG GCA TGG ATG GCA TCA ATC AC-3′ |
|  | Reverse | 5′-ACT CTA GCT GTG AAG TCA GTG TCG-3′ |

1.9. ps-TNC-CM components analysis

To identify porteins from CM, 1 mL of the basal medium, 1 mL of the untreated CM and ps-CTNC-CM were each loaded onto human angiogenesis array kit (R&D systems, Inc., Minneapolis, Minnesota, USA). After blocking the array membrane with blocking buffer for 1 h and membrane washing, the each of CM and array detection antibody cocktail was mixed and added to the blocked membrane followed by overnight shaking incubation at 4 °C. After washing, streptavidin-HRP buffer was added to the membrane, and incubation was performed for 30 min. Following another washing, Chemi Reagent Mixture was added to the membrane for reaction at room temperature and measured using LAS-3000 system (Fujifilm, Tokyo, Japan).
